# Mitochondrial oxygen monitoring with COMET: verification of calibration in man and comparison with vascular occlusion tests in healthy volunteers

**DOI:** 10.1007/s10877-020-00602-y

**Published:** 2020-10-21

**Authors:** R. Ubbink, M. A. Wefers Bettink, W. van Weteringen, E. G. Mik

**Affiliations:** 1 Department of Anesthesiology, Erasmus MC, University Medical Center Rotterdam, Rotterdam, The Netherlands; 2 Department of Pediatric Surgery, Erasmus MC—Sophia Children’s Hospital, University Medical Center Rotterdam, Rotterdam, The Netherlands

**Keywords:** COMET, Diagnostics, Mitochondrial oxygen tension (mitoPO2), PpIX-TSLT, 5-aminolevulinic acid

## Abstract

Mitochondria are the primary consumers of oxygen and therefore an important location for oxygen availability and consumption measurement. A technique has been developed for mitochondrial oxygen tension (mitoPO_2_) measurement, incorporated in the COMET. In contrast to most textbooks, relatively high average mitoPO_2_ values have been reported. The first aim of this study was to verify the validity of the COMET calibration for mitoPO_2_ measurements in human skin. The second aim was to compare the dynamics of mitoPO_2_ to several other techniques assessing tissue oxygenation. Firstly, we performed a two-point calibration. Mitochondrial oxygen depletion was achieved with vascular occlusion. A high mitoPO_2_ was reached by local application of cyanide. MitoPO_2_ was compared to the arterial oxygen partial pressure (PaO_2_). Secondly, for deoxygenation kinetics we compared COMET variables with the LEA O2C, SenTec OxiVenT™ and Medtronic INVOS™ parameters during a vascular occlusion test. 20 healthy volunteers were recruited and resulted in 18 datasets (2 times 9 subjects). The lowest measured mitoPO_2_ value per subject had a median [IQR] of 3.0 [1.0–4.0] mmHg, n = 9. After cyanide application the mitoPO_2_ was 94.1 mmHg [87.2–110.9] and did not differ significantly (n = 9, *p* = 0.5) from the PaO_2_ of 101.0 [98.0–106.0] mmHg. In contrast to O2C, OxiVenT™ and INVOS parameters, mitoPO_2_ declined within seconds with pressure on the probe. The kinetics from this decline are used to mitochondrial oxygen consumption (mitoVO_2_). This study validates the calibration of the COMET device in humans. For mitoVO_2_ measurements not only blood flow cessation but application of local pressure is of great importance to clear the measurement site of oxygen-carrying erythrocytes.

## Introduction

Mitochondria are small intracellular organelles that generate energy for the cells in the form of adenosine triphosphate (ATP). Oxygen is of critical importance for efficient ATP generation through the process of oxidative phosphorylation, also called mitochondrial respiration. This function makes mitochondria the primary consumers of oxygen in the body and therefore the most desired location for measuring oxygen availability and consumption.

An optical noninvasive technique has been developed for measuring mitochondrial oxygen tension (mitoPO_2_)_._ MitoPO_2_ is determined with the protoporphyrin IX-Triplet State Lifetime Technique (PpIX-TSLT) by measuring the oxygen-dependent delayed fluorescence lifetime of 5-aminolevulinic acid (ALA)-induced PpIX [1–3]. This measurement technique is incorporated in a medical device called the Cellular Oxygen METabolism monitor (COMET) [4].

Previous studies that used the protoporphyrin IX lifetime technique for cutaneous mitoPO_2_ measurements in humans reported some remarkable results. Most importantly, relatively high average mitoPO_2_ values of around 44 mmHg (5.9 kPa) [5] and 66 mmHg (8.8 kPa) [6] have been reported. In contrast, most textbooks mention normal values of mitochondrial oxygen tension as low as 7.5 mmHg (1 kPa) or less [7]. The calibration constants used in the COMET have been determined in animal studies [8]. A direct calibration in man has been lacking to preclude the high PO_2_ values being a result of improper calibration. The first aim of this study was therefore to verify the calibration of COMET in human skin.

No other clinical device is able to measure oxygenation at the mitochondrial level at the bedside. A direct comparison with other measurement techniques is thus unreliable because every tissue compartment, from intravascular to intracellular, has a different oxygen tension, leading to oxygen gradients. Due to the lack of a gold standard we aimed at using the same approach as used for in vivo calibration in animals, i.e. to use a combination of blocking oxygen supply by microvascular occlusion and blocking mitochondrial respiration by cyanide cream [8]. This provides a two-point calibration with a minimal mitoPO_2_ value during microvascular occlusion and a known mitoPO_2_ value after blockage of mitochondrial oxygen consumption.

Next to mitoPO_2_ measurements, the COMET system can be used to assess the parameters mitoVO_2_ (a measure for oxygen consumption) and mitoDO_2_ (as a measure for oxygen delivery) [3, 6]. Several methods have been developed over the years for measuring tissue oxygen consumption non-invasively. Most of these methods use hemoglobin-based measurement techniques, measuring a vascular or microvascular hemoglobin oxygen saturation in combination with a vascular occlusion test [9]. Typically, the mitoVO_2_ measurements with COMET show much faster deoxygenation kinetics than those other approaches [5]. Therefore, the second aim of this study is to compare COMET variables to spectroscopic and transcutaneous techniques during vascular occlusion testing.

In short, in this study we compare mitoPO_2_ with an arterial blood gas to verify the validity of the COMET calibration for mitoPO_2_ measurements in human skin and compared the dynamics of mitoPO_2_ to several other techniques for assessing tissue oxygenation in a series of healthy volunteers.

## Methods

The study was approved by the local medical ethical committee and registered on www.toetstingonline.nl [NL61767.078.17]. The study complies with the Helsinki declaration on research ethics. Healthy volunteers were recruited at Erasmus Medical Center Rotterdam, the Netherlands. Informed consent was obtained prior to participant inclusion. Inclusion criteria were: subjects between 18 and 50 years of age and ASA-1-2. Exclusion criteria were: mental disability, presence of mitochondrial disease, diabetes, anemia, hemoglobinopathy, mild to severe COPD, porphyria and/or use of anti-coagulant medication.

### Measuring mitochondrial oxygen tension

The COMET (Photonics Healthcare BV, Utrecht, The Netherlands) was used for mitoPO_2_ and mitoVO_2_ measurements. COMET uses the protoporphyrin IX triplet state lifetime technique (PpIX-TSLT) to measure oxygen availability. It provides quantitative measures, does not affect the measured tissue, and does not need recalibration before use [1]. For the extensive description of COMET internal components and the implemented algorithm, we refer to previous work [4]. We have described the fundamental principles behind the technology and have provided a working implementation of the technique as well as a method for calculating mitoVO_2_ from the mitoPO_2_ kinetics [10].

Before mitoPO_2_ measurements can be performed 5-Aminolevulinic acid (ALA) has to be applied to the skin to induce sufficient mitochondrial PpIX for detection of delayed fluorescence [1, 11]. To this end we cutaneously applied a 4 cm^2^ plaster, containing 8 mg ALA (Photonamic, Hamburg, Germany). 6–8 h previous to the measurements the ALA-plaster was applied to the lower arm. The COMET Skin Sensor was fixated onto the skin using a double-sided adhesive transparent plaster without optical interference (LEA Medizintechnik GmbH, Giessen, Germany).

### Verification of COMET calibration

Since no gold standard exists to which COMET can be compared, verification of COMET calibration had to rely on creating predictable mitochondrial oxygen levels. We chose a two-point verification aiming at approximating zero oxygen conditions and arterial oxygen tension. In earlier experiments in cells and animals the oxygen tension was decreased by flushing or breathing nitrogen to wash out all available oxygen.

In healthy human volunteers tissue-deoxygenation with nitrogen to a near-zero level is not a safe and viable option. A method that is applicable in humans is temporal arterial occlusion of a limb in combination with local pressure on the measuring probe. Arterial and microvascular occlusion inhibits blood flow and thus the oxygen supply to the measurement site. Ongoing cellular oxygen consumption will decrease local mitochondrial oxygen tension to very low values, approximating the desired zero oxygen conditions.

In addition to measurements near zero oxygen conditions we applied a method to compare mitoPO_2_ to arterial oxygen tension in a blood gas sample, in order to create a second calibration point at a higher PO_2_ level. A known high mitochondrial oxygen tension can be achieved by abolishing the oxygen gradient between arterial blood and the tissue cells. After cessation of mitochondrial oxygen consumption diffusion equilibrates the mitochondrial and arterial oxygen tension. Mitochondrial respiration can be temporarily inhibited by locally applying cyanide [12–14], which has previously been demonstrated in cells and animals [8]. In the transient absence of mitochondrial oxygen metabolism, the measured mitoPO_2_ can be compared to the oxygen partial pressure measured in an arterial blood gas (ABG) sample [12–14].

**Fig. 1:**
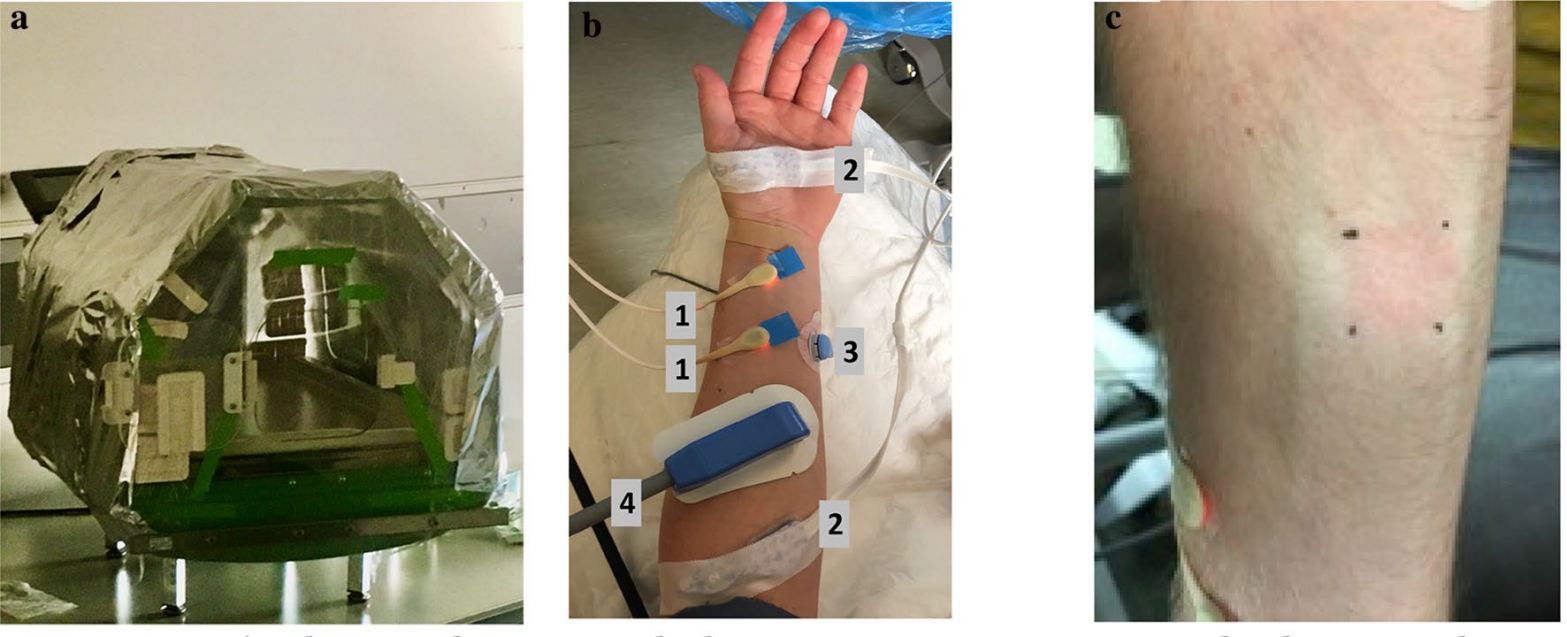
**a** The incubator, sealed to prevent nitrogen gas leakage and provide a controlled air temperature of 37 °C. **b** Probe position of 1. O2C LFX-43 probe, 2. INVOS, 3. SenTec OxiVenT, and 4. COMET Skin Sensor on the lower arm. **c** The ALA application side after cyanide application. A temporary hyperemia phase was seen as a red square on the arm

To diminish the influence of external factors like temperature and atmospheric oxygen a gas-sealed incubator was used to control internal air temperature and oxygen concentration, as shown in Fig. 1a. During the measurements the subject’s arm was inserted into the incubator, which was set to an internal temperature of 37 degrees Celsius. The oxygen concentration within the incubator was measured with a Fibox 4 trace (PreSense Precision Sensing GmbH, Regensburg, Germany). Prior to the mitoPO_2_ measurements the arterial blood pressure was taken.

The first mitoPO_2_ measurement was done in the incubator at a low surrounding PO_2_ achieved by filling the incubator with nitrogen gas (PO_2_ < 5 mmHg), and with the blood pressure cuff pressurized to 50 mmHg above systolic pressure. After verification of the cessation of blood flow with an O2C laser-doppler monitor with an LFX-43 probe (oxygen to see version 2424, Lea Medizintechnik GmbH, Germany), local pressure was applied with the measurement probe of the COMET to empty and occlude the microvessels in the measured tissue in order to perform a dynamic measurement showing the decreasing mitoPO_2_ (120 measurements at 1 Hz). With the combination of flow cessation with the pressure cuff, local pressure on the sensor, and mitochondrial oxygen consumption the minimal mitoPO_2_ was determined in the arm during occlusion. The lowest measured mitoPO_2_ during this measuring sequence was taken as lowest measurable mitoPO_2_ per subject.

An arterial blood sample was taken from the radial artery to determine arterial oxygen tension (PaO_2_) with a blood gas analyzer (ABL 800 Flex, Radiometer, Brønshøj, Denmark). Nitrogen gas was mixed with room air to set the oxygen concentration in the incubator to a level equal to the arterial PaO_2_ (range ± 5 mmHg).

In this study, topical application of cyanide was used to equalize mitoPO_2_ to PaO_2_ by blocking oxygen consumption at the skin measurement location. Cyanide ions (CN) bind with high affinity to the mitochondrial cytochrome c oxidase, blocking its activity. As a result, electron transport in the enzyme chain of oxidative phosphorylation and subsequently mitochondrial ATP production and mitochondrial oxygen metabolism are inhibited [15]. The applied cyanide cream was locally produced and contained a concentration of 1% potassium cyanide (Sigma-Aldrich, St. Louis, Missouri) mixed with hydrophilic cremor Lanette (Lanette cream I FNA, Bipharma, Weesp, The Netherlands). After 1 min the cream was removed and the lower arm was placed in the air mixture equal to the sampled PaO_2_, after which the mitoPO_2_ was measured.

To test blockage of mitochondrial respiration after topical application of cyanide the absence of oxygen consumption was checked. After 20 measurements in a sequence of 120 measurements at 1 Hz pressure was applied to the COMET skin sensor. Without cyanide a decrease in mitoPO_2_ within seconds was seen, as illustrated in Fig. 6a. While the mitochondrial respiration was blocked the mitoPO_2_ remained constant as illustrated in Fig. 6c. Fifteen minutes after cyanide application the mitoVO_2_ was measured in the skin to assess recovery of mitochondrial respiration (Fig. 6d).

### Comparison during vascular occlusion testing

To compare the behavior of mitoVO_2_ of COMET to other oxygen metabolism-related measurements, a variety of clinical bedside monitoring devices with a temporal resolution of seconds were used. The following devices were included; (1) Oxygen To See with the LFX-43 probe (O2C version 2424, Lea Medizintechnik GmbH, Germany), which combines direct (infra)red light spectroscopy with laser doppler. It measures local capillary venous saturation (SO_2_), and local microvascular blood flow is provided in flow units (FU). (2) Near-infrared spectroscopy (INVOS), which measures the tissue saturation and (3) a SenTec Digital Monitoring System with an OxiVenT™ Sensor (SenTec AG, Therwil, Switzerland) which transcutaneously measures blood gases and provides tcPCO_2_ values. The location of the different probes on the arm can be seen in Fig. 1b.

We measured during and after an arterial occlusion test of the arm. Arterial occlusion was achieved by insufflation of a cuff to at least 50 mmHg above the systolic blood pressure. The absence of skin blood flow was confirmed with laser-doppler blood flow measurement with the O2C. During 2 min the COMET monitor measured mitoPO_2_ with a frequency of 1 Hz. The O2C, INVOS, and SenTec OxiVenT all collected data during arterial occlusion to determine the oxygen level and consumption, as well as carbon dioxide (CO_2_) accumulation in the measurement volume. In this setup no pressure was applied to the COMET skin sensor in order to be able to compare the deoxygenation rates of the different measurements.

The mitoVO_2_ measurement was done with the following procedure; after a stationary measurement of 10 s direct pressure on the COMET Skin Sensor probe was applied. This halted the microcirculation, and with it oxygen delivery to the measured tissue volume. The mitoVO_2_ was measured directly after local occlusion of the oxygen supply by a linear fit of mitoPO_2_. This simple procedure created reproducible stop-flow conditions and induced measurable oxygen consumption rates, consequential to a cessation of microvascular oxygen supply and ongoing cellular oxygen consumption. MitoPO_2_ was measured before, during and after application of pressure at an interval of 1 Hz.

### Statistical analysis and software

Software version v.016.5b.184 of COMET was used, during the cyanide measurements, the adjusted timing software was used. Statistical analysis and visualization were done with R version 3.4.2 [16] and GraphPad Prism 6. MitoPO_2_ and Arterial Blood Gas were compared using a two sided Wilcoxon-Mann–Whitney U-test. Significance was determined by p-values < 0.05. Values are given as median and interquartile ranges or stated otherwise.

The average of the last 3 MitoPO_2_ values measured before pressure was applied were used as a baseline. Linear slope comparison (as measure for the deoxygenation rate, or oxygen disappearance rate) between COMET, INVOS, SenTec OxiVenT was performed with GraphPad Prism linear regression model from the descending part of the data. MitoVO_2_ linear fit was done with LabVIEW (Version 13, National Instruments, Austin, TX, USA) with the first 4 samples after pressure had been applied by the sensor to compare mitoVO_2_ with previous published results [5]. To visually compare the oxygenation decline rate between the devices the data is transformed into z-score (datapoint − mean)/standard deviation.

## Results

**Fig. 2:**
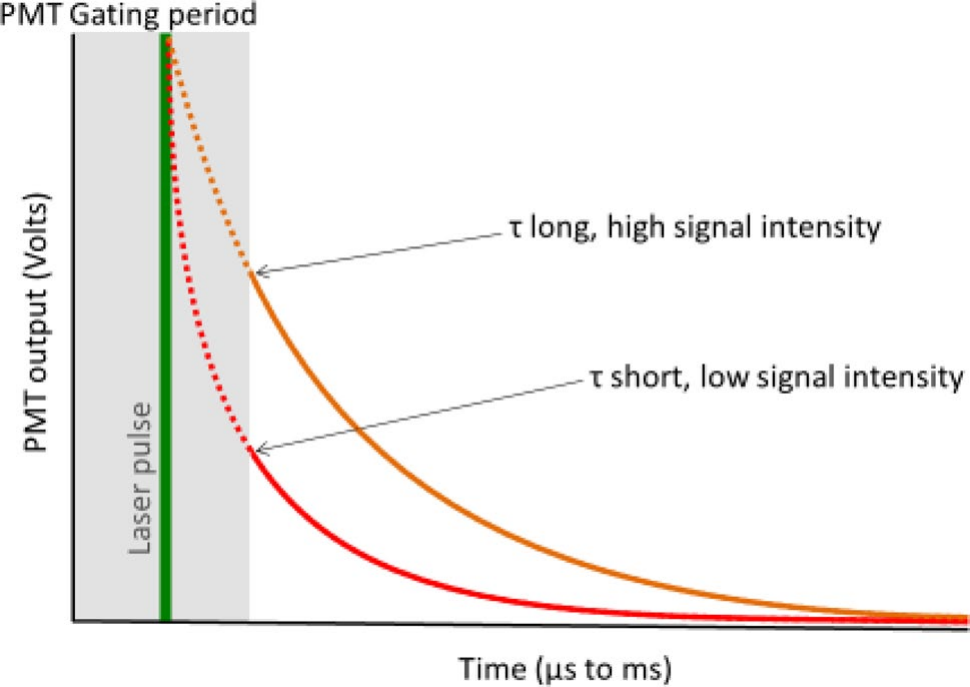
This illustration shows the decrease in signal intensity measured by the photomultiplier (PMT) with short lifetimes (several μs). If the lifetime is short the oxygen tension is high. The laser pulse (green) is followed by a PMT gating period (gray) to protect the PMT from prompt fluorescence. At the PMT most of the short lifetime signal intensity (red) will be lost. In low oxygen tension and thus a long lifetime (orange) the signal intensity will hardly be influenced by the PMT gating period

A total of 20 healthy volunteers were recruited and provided informed consent. Of the 20 volunteers 10 underwent the entire study protocol. One subject dropped out due to beta-thalassemia that was missed during the inclusion procedure. The first 10 inclusions (session 1) resulted therefore in 9 complete datasets. Analysis of this first dataset showed that cyanide application led to unmeasurable delayed fluorescence in all but 1 subject. This unforeseen result was analyzed in cooperation with the manufacturer of the COMET device. It appeared to be caused by the relatively long photomultiplier gate duration in comparison to the very short delayed fluorescence lifetimes after cyanide application, illustrated in Fig. 2.

**Fig. 3:**
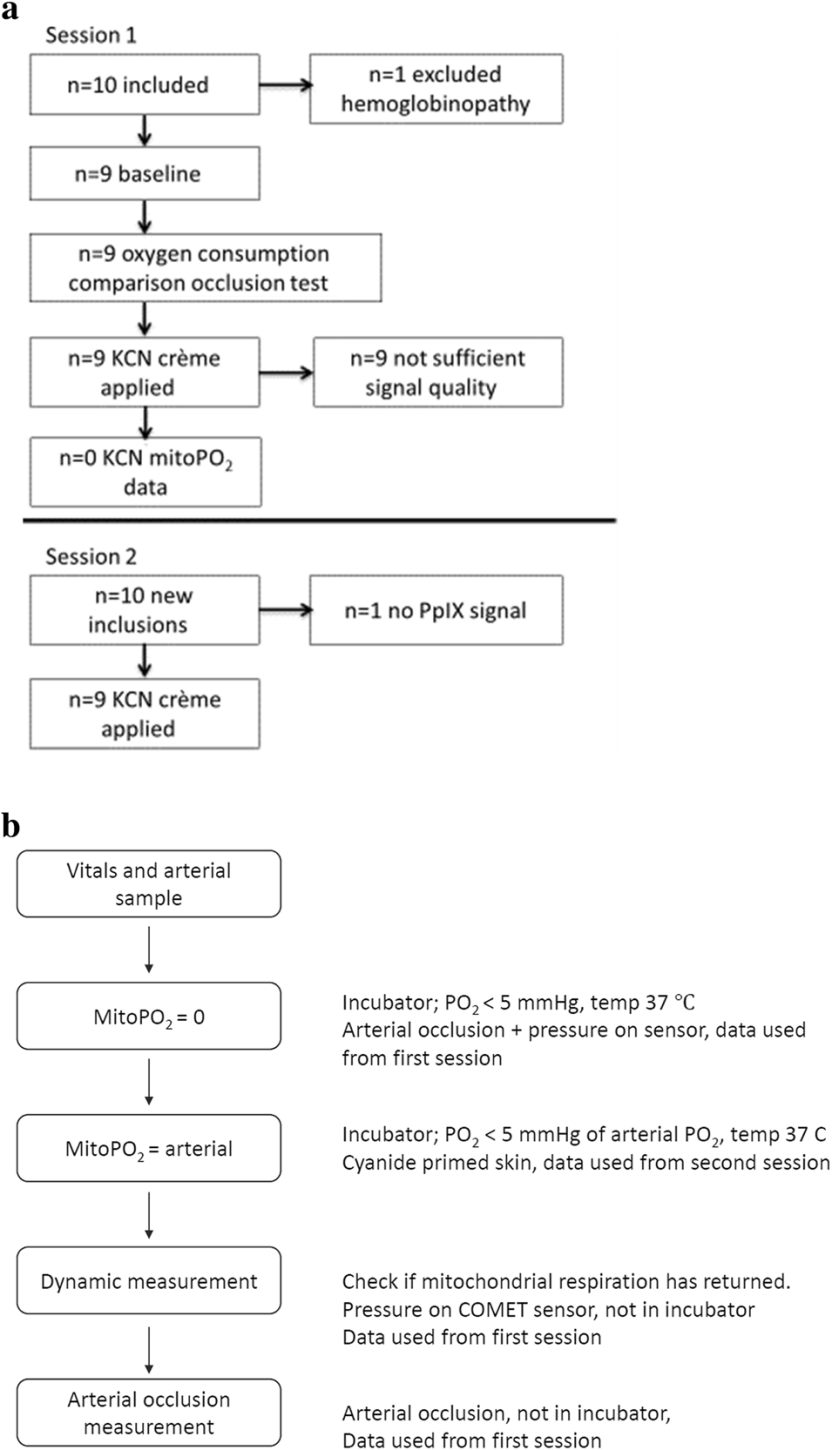
**a** Diagram of study subject flow, **b** Diagram of methods timeline

A temporary change in the firmware of the COMET, kindly supported by the manufacturer, was suggested and used to overcome this problem. Therefore, a second series of measurements (session 2) with the cyanide cream was performed in 10 subjects. One subject had insufficient signal quality after the baseline measurement. This resulted in 9 datasets of new volunteers in the second session. A flow diagram of the inclusions is shown in Fig. 3.

Demographic characteristics of the 18 analyzed healthy volunteers can be found in Table 1.

**Table 1:** Volunteer characteristics

	Session 1 (n = 9) Median [IQR]	Session 2 (n = 9) Median [IQR]
Plaster application time (min)	365 [360–450]	434 [425–455]
Blood pressure systole (mmHg)	123 [120–125]	129 [124–130]
Blood pressure diastole (mmHg)	84 [78–85]	85 [80–90]
Body length (cm)	184 [173–190]	178 [173–181]
Weight (kg)	84 [65–90]	77 [71–80]
Age (years)	28 [26–30]	28 [23–32]
Gender (female)	22%	33%

### Zero and arterial blood gas oxygen tension validation

**Fig. 4:**
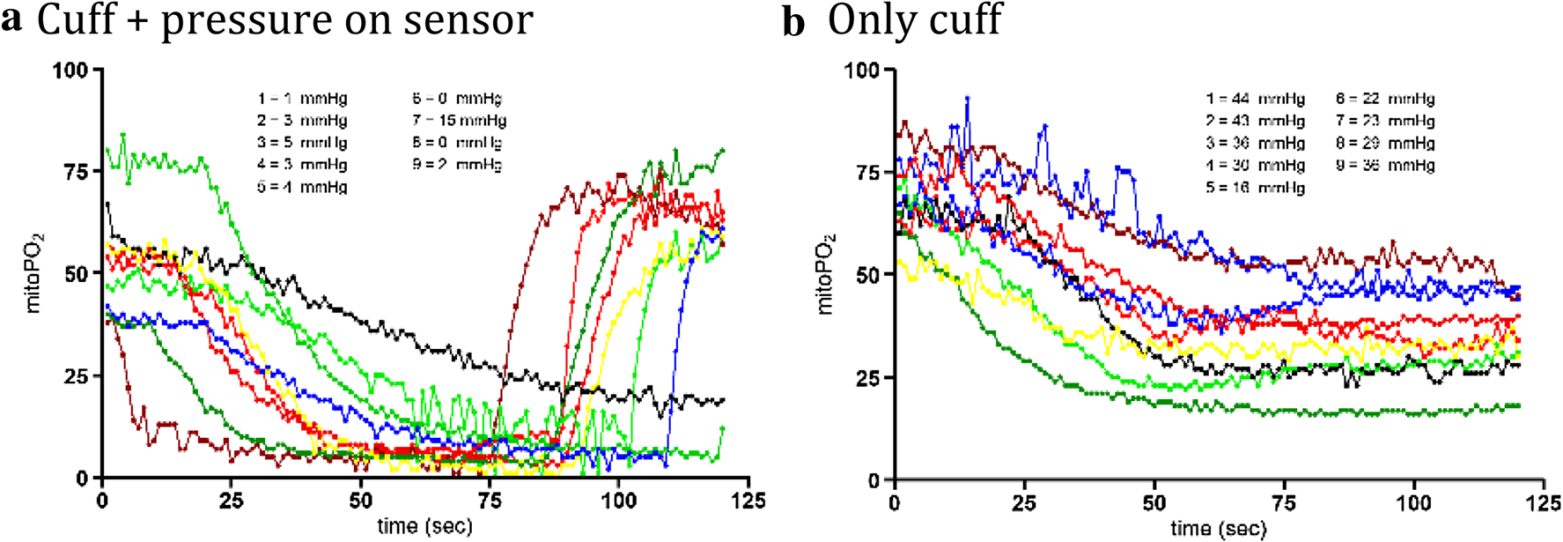
**a** mitoPO_2_ measurements in the incubator with pressure applied to the COMET skin sensor and an inflated upper arm cuff, pressurized to 50 mmHg above systolic blood pressure to stop the arterial flow in the arm. The listed values are the individual minimum mitoPO_2_ levels just before the pressure is released. **b** mitoPO_2_ measurements of the arm in which only the cuff around the upper arm was pressurized to 50 mmHg above systolic blood pressure to stop the arterial blood flow in the arm. The listed mitoPO_2_ values are the equilibrium value at the end of the measurement

While the arm was in the incubator with an oxygen tension < 5 mmHg, the blood supply to the arm was occluded and pressure was applied to the skin sensor to approximate zero oxygen conditions. In all cases mitoPO_2_ dropped and reached an equilibrium. The lowest measured mitoPO_2_, median [IQR] minimum value was 3.0 [1.0–4.0] mmHg. In one case the steady state did not go below 5 mmHg and reached an equilibrium at 15 mmHg, seen in Fig. 4a.

The additional effect of local pressure on the tissue with the COMET skin sensor on the deoxygenation kinetics compared to use of only a blood pressure cuff can clearly be seen in Fig. 4. In the experiments shown in Fig. 4b only an upper arm cuff was pressurized to stop the arterial blood flow. As a result, the mitoPO_2_ stabilized at median of 30.0 [24.5–36.0] mmHg, greatly contrasting with the low mitoPO_2_ 3.0 [1.3–4.8] mmHg when pressure is also applied on the skin sensor itself. If pressure is applied to the skin sensor the decrease in oxygen tension is faster, 2.1 [1.0–2.9] mmHg/s compared to 1.3 [1.2–1.4] mmHg/s without pressure.

**Fig. 5:**
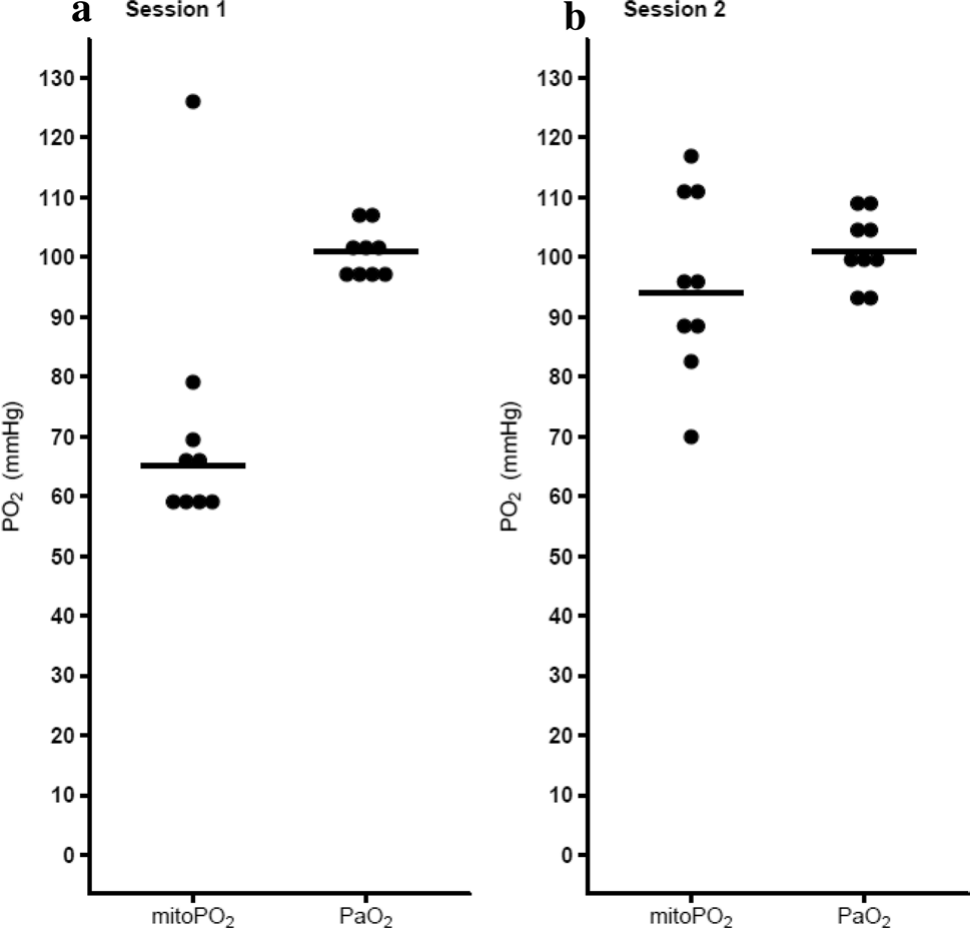
Comparison between COMET mitoPO_2_ after cyanide cream had been applied to block mitochondrial respiration and arterial blood gas PaO_2_ taken from a radialis. **a** Data from session 1, even though the delayed fluorescence was insufficient a mitoPO_2_ was displayed around 65 mmHg. One subject with a strong signal the short lifetime was measurable, seen as black dot 126 mmHg. **b** Data from session 2 with adjusted software. No significant difference was seen in session 2 between mitoPO_2_ and PaO_2_

Application of cyanide cream on the skin in the first session led to low signal quality and, except for one case, mitoPO_2_ readings well below the corresponding PaO_2_ values (Fig. 5a). This COMET behavior was analyzed in cooperation with the manufacturer and appeared to be caused by the detector gating. Due to the specific timing of this gating, the very short delayed fluorescence lifetimes resulting from the high intracellular PO_2_ were omitted from the signal analysis, leading to an erroneously low steady mitoPO_2_ around 66 mmHg.

**Fig. 6:**
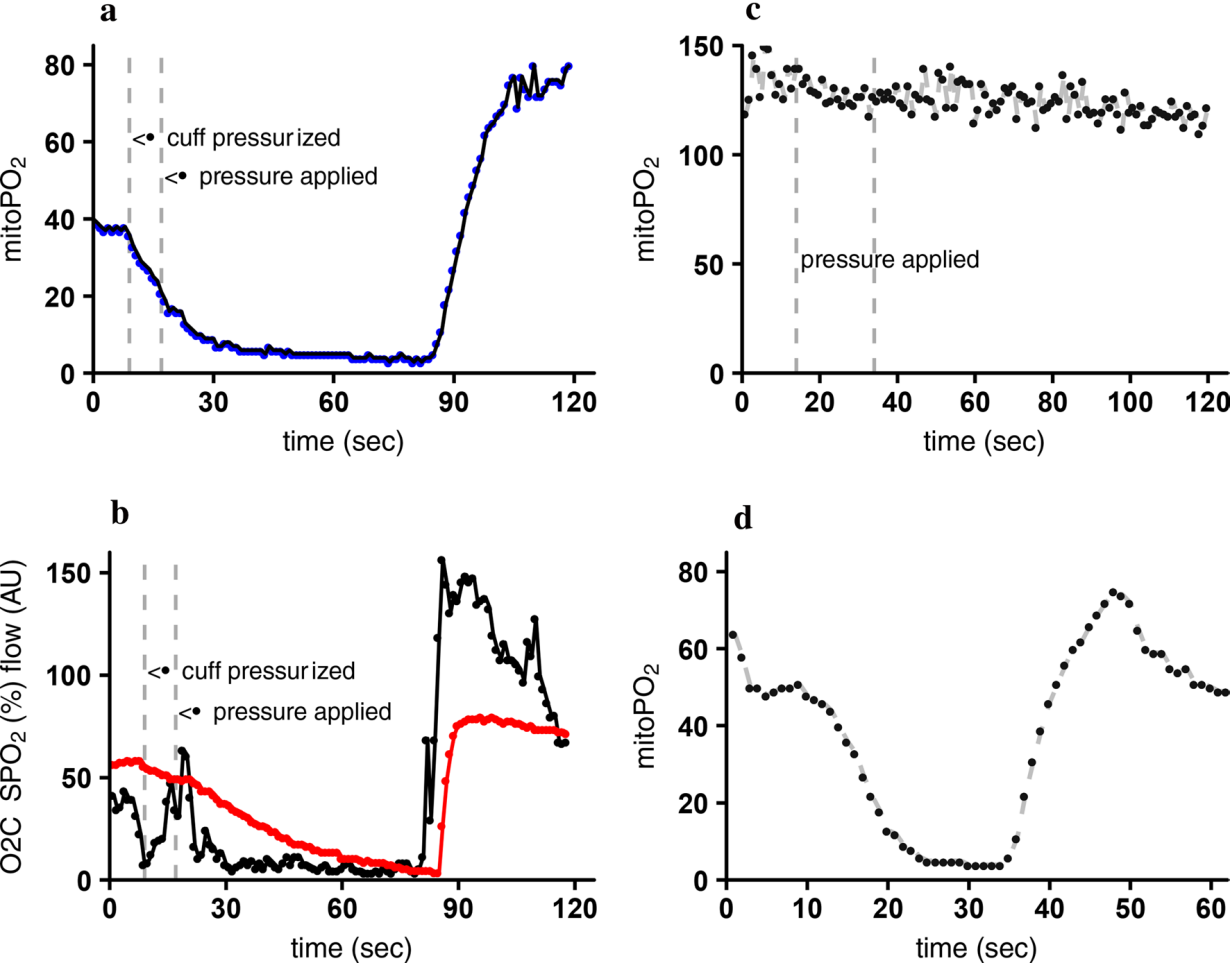
**a** MitoPO_2_ baseline measurement, at t = 9 (sec) blood pressure cuff inflated to 50 mmHg above systolic blood pressure, t = 17 (sec) manual pressure on the COMET skin sensor, at t = 84 (sec) release of pressure from skin sensor and cuff. **b** O2C microvascular blood flow (black) and tissue oxygenation SpO_2_ (red) with the pressure cuff applied and pressure on the skin sensor. **c** Cyanide was applied to the skin to block mitochondrial respiration. While pressure was applied no sudden drop was seen, indicating cyanide-induced blockage of mitochondrial respiration. **d** Ten minutes after cyanide application a mitochondrial respirational check was done

To enable accurate detection of high mitoPO_2_ values the timing of the gating was adjusted by a temporary adaptation in the firmware. During session 2 data was collected with this adjusted software. Now, the median [IQR] mitoPO_2_ was 94.1 mmHg [87.2–110.9] and did not differ significantly (*p* = 0.5) from the sampled PaO_2_ of 101.0 mmHg [98.0–106.0]. When pressure was applied to the skin sensor the mitoPO_2_ did not decrease, indicating the absence of mitochondrial respiration, as shown in Fig. 6c. Within all subjects the mitochondrial respiration returned after approximately 15 min. No major adverse events caused by the cyanide application were witnessed. Apart from a temporarily red skin, no pain, skin irritation or other effects of the cyanide cream were reported.

### Comparison of monitors

**Fig. 7:**
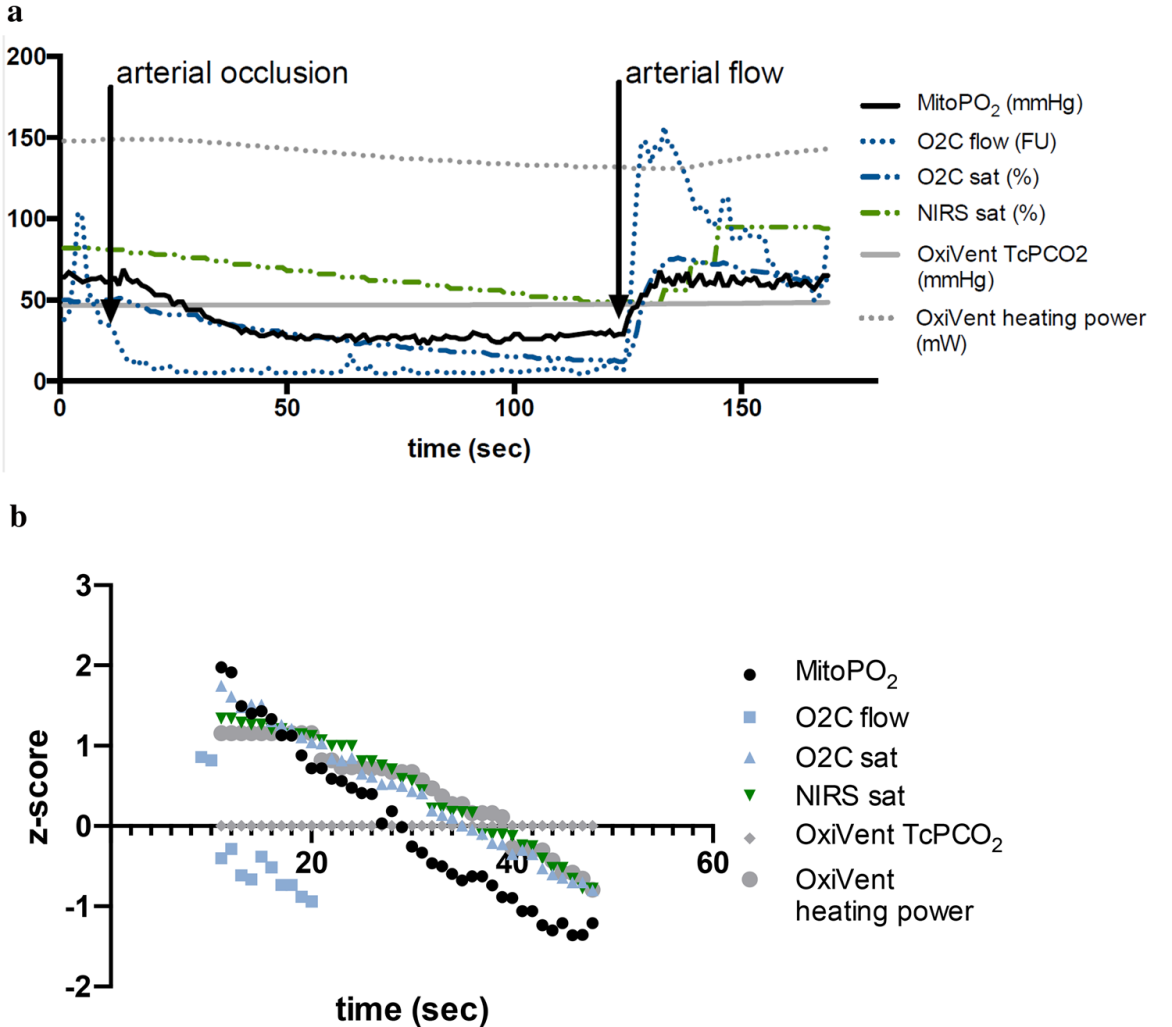
**a** Typical example of arterial occlusion of the forearm. At t = 10 s the blood pressure cuff was inflated to 50 mmHg above the blood pressure measured earlier. At t = 120 s the blood pressure cuff was released and arterial flow returned. **b** Average data of 9 subjects. Data shown in z-score transformation, z = (data point − mean)/standard deviation to compare the overall decline part of the data

Arterial occlusion of the arm led to an immediate decline and subsequent stop of microcirculatory blood flow measured by the O2C. MitoPO_2_ and tissue oxygen saturation followed. A linear regression of the measured decline during the arterial occlusion with the cuff provided different slopes for all measurements as shown in Table 2. An example of measurements is shown in Fig. 7a, with the mean of the z score of all subjects shown in Fig. 7b.

**Table 2:** Linear fit of arterial occlusion of the arm with a pressurized cuff

	Baseline value Median [IQR]	Decline Mean ± SD	z-score* Decline Mean
MitoPO_2_ (mmHg)	68 [61–76.5]	− 0.75 ± 0.06	− 0.089
Flow O2C (FU)	35 [20–57]	− 2.30 ± 0.37	− 0.15
sat O2C (%)	49 [43–65]	− 0.51 ± 0.05	− 0.062
NIRS (%)	76 [72–82]	− 0.21 ± 0.04	− 0.058
TcPCO_2_ OxiVenT (mmHg)	51 [47–56]	− 0.37*10^–3^ ± 0.03	− 0.010
Heating OxiVenT (mWatt)	130 [123–142]	− 0.95*10^–1^ ± 0.06	− 0.050

*z-score = (data point − average)/standard deviation

Interestingly the tcPCO_2_ measurement was stable during this short arterial stop, although the heating power required to maintain a stable sensor temperature of 43 °C changed slightly. After a relatively long delay an increase in tcPCO_2_ was seen.

## Discussion

This study demonstrates that the calibration of COMET device, originally determined in animal experiments, is adequate for cutaneous mitoPO_2_ measurements in man. Furthermore, dynamic oxygenation measurements aimed at gaining insight in tissue oxygen consumption depend on the specific measuring technique and method to cease oxygen supply to the measurement spot.

The COMET measurement system measures relatively high mitoPO_2_ values compared to the general idea that mitoPO_2_ should be very low in order to drive oxygen diffusion [6, 17, 18]. This study shows that such high mitoPO_2_ values are not the result of an inadequate calibration. On the contrary, in this study the COMET showed the tendency to underestimate mitoPO_2_ in the case intracellular PO_2_ was artificially increased to arterial oxygen levels.

The mitoPO_2_ measured with the COMET monitor is higher than expected. MitoPO_2_ appears to be, depending on the measurement site and respiratory rate of the tissue, much closer to microvascular oxygen tension [19, 20], and thus closer to tissue and/or interstitial oxygen levels [21, 22], than anticipated [7]. There are several reasons why mean mitoPO_2_ in a tissue sample cannot be an order of magnitude lower than microvascular and interstitial oxygen tension; first, oxygen does not disappear stepwise but gradual, so several mitochondria will see aPO_2_ close to intravascular values. Second, oxygen diffuses also from large vessels so contribute to cellular oxygen delivery [23], so several mitochondria have a higher PO_2_ than capillary oxygen tension. Third, the mitoPO_2_ will not be substantially lower than interstitial PO_2_ because the oxygen gradient over the cell membrane is small [1]. Typically reported baseline mitoPO_2_ values are 40–70 mmHg. Other oxygen measurements in the skin are matching these values [22]. Importantly, it has been demonstrated in both a preclinical [24] and clinical setting [4] that mitoPO_2_ provides different information than hemoglobin saturation-based techniques like near-infrared spectroscopy.

Application of cyanide on the skin led to a temporary block of mitochondrial respiration and abolishment of the oxygen gradient. In the first series of 9 investigated subjects, the timing between the laser pulse and the end of the off-gating of the photomultiplier (PMT) in the COMET proofed too long to adequately detect the short delayed fluorescence lifetimes caused by the artificially high intracellular PO_2_. The gating itself is necessary to prevent damage to the sensitive detector due to laser light and prompt fluorescence [4], and its timing is a trade-off between several factors, foremost the ability to accurately measure high mitoPO_2_ (supraphysiological) and protecting the detector. After adjustment of this timing in the firmware, PMT gating interference was sufficiently reduced to allow collection of the delayed fluorescence signal after topical application of cyanide. Due to this adaptation, we were able to demonstrate that mitoPO_2_, as measured with COMET, corresponds well to PaO_2_ in the absence of mitochondrial oxygen consumption. Under more physiological circumstances the timing of the PMT gating is much less critical as delayed fluorescence lifetimes are longer and easier to detect. COMET measured very low mitoPO_2_ after oxygen deprivation and overall the calibration of the device seems adequate for its purpose.

This study also presents the comparison of different oxygen consumption measurements in the arm. The COMET was compared to O2C, INVOS and SenTec OxiVenT™ during an arterial occlusion test in nine subjects. During arterial occlusion all oxygenation parameters show a decline in a comparable rate. Also, the measured decline in NIRS saturation of 0.21%/sec (12.6%/min) in this study is comparable to previously found values of 10.8, 13.2, 22.8%/min during occlusion of an extremity [25]. However, during a dynamic measurement for measuring mitochondrial oxygen consumption (mitoVO_2_), with pressure on the measurement probe, a faster decline is seen. This decline is seen in all but one subject in Fig. 4a with an equilibrium at 15 mmHg. Since the curve of this individual shows similarities to the measurements without local pressure on the probe, we think that probably the effect of local pressure on the sensor was inadequate. We hypothesize that in this case an equilibrium emerges between the still saturated hemoglobin and mitochondrial respiration, similar to the situation in laboratory animals [26]. Also, when a mitoVO_2_ procedure is done on the sternum we do not see such equilibrium at a high mitoPO_2_ value. Therefore, we think that the pressure on the skin sensor did not adequately push away the erythrocytes in the measurement volume.

MitoVO_2_ measurements should preferably be done on skin above a bone structure to allow the buffer of erythrocytes to be pushed away. The arm is therefore not the preferred site because the skin is not located above a flat bone. This likely resulted in a relatively slow median mitoVO_2_ of 2.1 mmHg/s in comparison to measurements done on the sternum with a median mitoVO_2_ 5.8 mmHg/s measured on healthy volunteers in our lab [5]. When the skin sensor is on top of a bone structure, with a little pressure the microcirculation is blocked and the erythrocytes are pushed out of the measurement volume. In this study the applied pressure was not measured but this could add to the standardization of a mitoVO_2_ maneuver and improve the repeatability. However, arterial occlusion tests can only be done on an extremity and therefore the mitoVO_2_ values are different from other healthy volunteer studies [5, 6]. Whilst the forearm is not a preferred measurement site a large difference in mitoVO_2_ could be demonstrated if pressure is exerted onto the COMET Skin Sensor, 2.1 mmHg/s compared to 1.3 mmHg/s during arterial occlusion. The oxygen buffer available in microcirculation likely accounts for the difference of 0.8 mmHg/s.

Both the mode of measurement (hemoglobin-based versus non-hemoglobin-based) and differences in tissue penetration depth per technique might account to the observed differences in oxygen disappearance rates. The COMET has a penetration depth of less than a mm, in contrast to infra-red optical techniques (> 900 nm wavelength) with a penetration depth of several cm. Since O2C and INVOS measure deeper in the tissue, and thus in a different tissue compartment, the decline in oxygen saturation represents a larger measurement volume. After the occlusion an equilibrium emerges between the available oxygen, mainly dependent on the concentration and amount of arterial and venous hemoglobin available in the vessels, and mitochondrial respiration. It is not possible to push away or largely reduce the number of erythrocytes in the infra-red measurement volume. A large measurement volume that contains erythrocytes without the ability to reduce the oxygen buffer results in a slower decrease in saturation. When performing a dynamic measurement with the COMET local pressure with the Skin Sensor leads to largely eliminating the available erythrocytes from the microcirculation and therefor the dynamic measurement may be less dependent on the availability of the oxygen buffer.

## Conclusion

This study shows that mitochondrial oxygen partial pressures measured with Pp-IX lifetime technique are comparable to the arterial PaO_2_ during blockade of mitochondrial respiration with topical application of cyanide. Therefore, this study demonstrates that the calibration of COMET device, originally determined in animal experiments, is valid in human cutaneous mitoPO_2_ measurements.

For mitochondrial oxygen consumption measurements not only blood flow occlusion, but applying pressure on the COMET Skin Sensor is of great importance to clear the measurement site of available oxygen-carrying erythrocytes. Without a technique to eliminate this oxygen buffer the consumption measurement underestimates the actual mitochondrial oxygen consumption.

## Data Availability

Additional data can be requested from the corresponding author.
